# Editorial: When Data Science, Humanities and Social Sciences Meet: Cross-Talks and Insights in Public Health

**DOI:** 10.3389/fpubh.2020.00041

**Published:** 2020-02-25

**Authors:** Thomas Lefèvre, Sandrine de Montgolfier

**Affiliations:** ^1^IRIS Institut de Recherche Interdisciplinaire sur les enjeux Sociaux (UMR 8156 CNRS - 997 INSERM - EHESS - UP13), Paris, France; ^2^Department of Legal and Social Medicine, Hôpital Jean Verdier, AP-HP, Bondy, France; ^3^UPEC (Université Paris Est Creteil) & Inspé, Créteil, France

**Keywords:** big data, public health, social science research, humanities, artificial intelligence

Big data is still an emerging, fast-changing topic in medicine, but it originated in the older and larger field of data science. Data science is about collecting and merging data of various natures from diverse sources, but also has to do with mining data for information retrieval and providing observation-based support for decision-making (1). If we refer to broad definitions of health such as WHO's definition (2), Engels' biopsychosocial definition (3), or even the syndemics approach (4), we may wonder whether data science could be seen as a way of looking at health in a very comprehensive and multidimensional way by integrating these multiple data analysis. It could be seen as a way to go beyond the limits of the evidence-based approach to health.

In this context, public health addresses an even broader range of challenges by taking into account from the (sub)individual to the societal and global scales to build its knowledge and ground its actions. As of today, by heavily relying on biomedical, quantitative techniques, the field of public health faces all kind of issues related to real life, individual, and social complexity.

This complexity is faced by social sciences and humanities (SSH) which historically had to prove that they were autonomous scientific fields, as Durkheim managed to do by grounding sociology as a science by defining the field's own scientific method (5). SSH researchers developed and tried many methodological approaches. As a result, SSH may be methodologically richer than the biomedical field, which has been so far mainly restricted to a certain variety of statistical methods. We may wonder how it would be possible to reconcile these two approaches to better approach the concept of health (6).

Data science applied to health and public health could lead to a genuine data-driven evidence-based policy making. The human sciences are then really necessary to question ethically, legally and socially the use of data science: when action is possible, should it be actually performed? Data science questions how and how far behavioral changes should be encouraged, how mass monitoring and control are to be authorized. SSH specialists are used to these questions and can provide critical clues. SSH researchers with their own methods for questioning our world will therefore have to imbue data science with new approaches and perspectives. Conversely, data science can enrich SSH toolbox with hybrid or novel quantitative methods. Finally, a “meet me halfway” strategy should be considered so that tools designed for both SSH researchers and data scientists are conceived. A series of cross-talks between data scientists and SSH researchers could provide valuable insights for public health.

The various contributions to this Research Topic dedicated to this type of a cross-talk bring us back to the practical essence of big data through several illustrations and points of view: big data consists primarily of building if not sewing a unique, larger database from distinct smaller databases. The new database is supposed to be homogenous by many aspects as if it had been constituted from one unique experience. Of course reality is different: each smaller, sewed database has been built from a specific experiment at a time and in a location that are unique to it. Questionnaires, regulations and laws that apply to the experiment, people behind the experiment… all of these aspects can vary by much from one experiment to another. Handling a big data collection is therefore different from handling a mere addition of smaller databases.

Social epidemiologists Delpierre and Kelly-Irving use the example of how big data can contribute to the study of social inequalities in health. They illustrate precisely the consequences of building and handling a big data collection made of a variety of smaller databases.

Sociologist Derbez grounds his argument on the experience of the breast cancer genetics. Data are routinely collected in a variety of contexts and questions arose about the property of data (public vs. private stakeholders) when several providers exist and about the information and participation to research of citizens and patients contributing to the database.

Sociologist and anthropologist Krikorian and Vailly investigate how ethical management of health data can inform the use of data collected about DNA in another context: the initial and secondary uses that police can make of DNA. In the same way that data are collected into a larger database, the ethical framework attached to the resulting big data collection appear to be a collection of local and *ad hoc* smaller ethical rules attached to each small database.

Data scientists Young et al. explain how data linkage e.g., with social media could help policy makers and researchers to assess the social and economic impact of health policy in near real time compared to traditional, time-consuming, and expensive studies.

Last but not least, voice is given here to future medical doctors and also data scientist apprentices. Medical student Lerner et al. share their point of view on how they imagine that the doctor-patient relationship and the medical doctor skills could and should evolve as data science is everyday more present in healthcare. They recall that healthcare practitioners must value all the human skills that machines and data are not likely to replace. Conversely, these practitioners should be informed, trained, and aware of what data science is and is not able to add to their medical activity.

## Author Contributions

TL and SM drafted the manuscript and contributed to its intellectual content.

### Conflict of Interest

The authors declare that the research was conducted in the absence of any commercial or financial relationships that could be construed as a potential conflict of interest.
